# Development and validation of a model for early diagnosis of biliary atresia

**DOI:** 10.1186/s12887-023-04370-x

**Published:** 2023-10-31

**Authors:** Zongrong Gong, Lin Lin, Gen Lu, Chaomin Wan

**Affiliations:** 1 Department of Pediatrics, West China Second Hospital, Sichuan University and Key Laboratory of Birth Defects and Related Diseases of Women and Children, Ministry of Education, No 20, 3rd Section of Renmin South Road, Chengdu, 610041 People’s Republic of China; 2 Department of Respiration, Guangdong Provincial Clinical Research Center for Child Health, Guangzhou Women and Children’s Medical Center, Guangzhou Medical University, Guangzhou, 510623 China; 3 Department of Pediatric Infectious Disease, West China Women’s and Children’s Hospital, Key Laboratory of Birth Defects and Related Diseases of Women and Children, Second University Hospital, Sichuan University, Ministry of Education, No 20, 3rd section of Renmin South Road, 610041 Chengdu, P.R. China

**Keywords:** Biliary atresia, Neonatal cholestasis, Diagnostic model

## Abstract

**Background and aims:**

Early diagnosis of biliary atresia (BA), particularly distinguishing it from other causes of neonatal cholestasis (NC), is challenging. This study aimed to design and validate a predictive model for BA by using the data available at the initial presentation.

**Methods:**

Infants presenting with NC were retrospectively identified from tertiary referral hospitals and constituted the model design cohort (*n* = 148); others were enrolled in a prospective observational study and constituted the validation cohort (*n* = 21). Clinical, laboratory, and abdominal ultrasonographic features associated with BA were assessed. A prediction model was developed using logistic regression and decision tree (DT) analyses.

**Results:**

Three predictors, namely, gamma glutamyl transpeptidase (γGT) level, triangular cord sign (TC sign), and gallbladder abnormalities, were identified as factors for diagnosing BA in multivariate logistic regression, which was used to develop the DT model. The area under the receiver operating characteristic (ROC) curve (AUC) value for the model was 0.905, which was greater than those for γGT level, TC sign, or gallbladder abnormalities alone in the prediction of BA.

**Conclusion:**

A simple prediction model combining liver function and abdominal ultrasonography findings can provide a moderate and early estimate of the risk of BA in patients with NC.

## Introduction

Neonatal cholestasis (NC) is a relatively common clinical issue that presents a complex diagnostic challenge for clinicians [1]. Cholestasis includes a complex set of aetiologies. Therefore, the identification of life-threatening and treatable causes of cholestasis is a high priority. Biliary atresia (BA), which has an overall incidence of approximately 1 in 8000 to 1 in 17,000 [2], can rapidly progress to biliary cirrhosis and hepatic failure, necessitating liver transplantation. BA is the most common cause of liver transplantation in children [3] and should be distinguished from other nonsurgical causes of cholestasis in a timely manner. At present, early Kasai portoenterostomy (KP) is associated with longer periods of survival without liver transplantation, and in neonates over 60 days of age, the jaundice disappearance rate decreased to 57.0% [4]. Some of the single methods reported in the literature, such as gamma glutamyl transpeptidase (γGT) and triangular cord sign (TC sign), can diagnose BA with moderate accuracy in an NC patient population [5, 6]; most of these methods were based on single studies, and their accuracy requires improvement. Although a few scoring systems have been proposed [7, 8], these involved invasive diagnostic methods, such as liver biopsy, and did not target patients within 2 months of age.

In this study, we aimed to develop and validate a simple and early predictive diagnosis model based on noninvasive diagnostic methods, including clinical, laboratory, and imaging data, to predict the risk of BA in patients with NC. The results of this study may offer a novel and better algorithm for the early diagnosis of BA and hold potential for clinical application.

## Patients and methods

### Human participants

This retrospective and prospective study included two consecutive cohorts of infant patients with NC (1041 in total) who were collected, reviewed, and analysed from the Department of Pediatrics of West China Second University Hospital, Sichuan University, China. Of these, 781 patients were aged over 60 days, 62 patients were assigned to the BA group, and 107 patients were assigned to the non-BA group. The first cohort (model design cohort) consisted of 148 infants between December 2008 and December 2017. The second cohort (validation cohort) consisted of 21 consecutively recruited infants with NC between January 2018 and December 2018. NC was defined by direct or conjugated bilirubin (DBIL) concentration > 17.1 µmol/L, if total serum bilirubin (TBIL) concentration ≤ 85.5 µmol/L, or DBIL concentration > 20% of the TBIL concentration, if TBIL concentration > 85.5 µmol/L [9]. The inclusion criteria were a diagnosis of NC and age ≤ 60 days when visiting our centre. The exclusion criteria were parental refusal of Kasai surgery; loss to follow-up; and other severe systematic deformities, such as BA splenic malformation syndrome.

### Diagnosis

After complete history-taking, thorough clinical examination, and routine investigation, the diagnosis of BA was confirmed by laparotomy and intraoperative cholangiography (IOC) prior to KP. A diagnosis of BA was ruled out on the basis of specific laboratory tests based on the expected aetiology, negative IOC results in some patients, and follow-up evaluations. Diagnosis in the validation cohort was confirmed as BA by IOC and as non-BA either by confirming the actual aetiology or by excluding the possibility of BA by IOC in cases where the aetiology could not be reached. Data from routine investigations, including stool colour assessment; measurement of TBIL and DBIL concentrations, direct bilirubin/total bilirubin ratio (DBIL/TBIL), alanine transaminase (ALT) concentration, aspartate transaminase (AST) concentration, alkaline phosphatase (ALP) concentration, total bile acid (TBA) concentration, lactate dehydrogenase (LDH) concentration, and gamma glutamyl transpeptidase (γGT) concentration; complete blood count; ultrasonography; and Doppler ultrasonography were reviewed.

### Ultrasonographic evaluation

Ultrasonography was performed using 9 − 3 MHz and 5 − 2 MHz Philips IU 22 and 12 − 5 MHz and 5 − 2 MHz Philips HD 11 (Royal Philips Electronics, the Netherlands). Combined low-frequency and high-frequency ultrasound was used to examine portal hepatic microcysts for strong echogenic fibrous masses and gallbladder abnormalities. Patients fasted for 4 h before examination and were re-examined 30 min after feeding to evaluate gallbladder contractility. Parameters assessed included the size of the liver, TC sign positivity and abnormal gallbladder. Liver enlargement was defined when the maximum oblique diameter of the right liver exceeded the upper limit of the normal age (90 mm) [10], the thickness of the echogenic anterior wall of the right portal vein just proximal to the right portal vein bifurcation site, which was used to identify the TC sign [11], and TC sign positivity was defined when the thickness of TC sign was over the cut-off value of the ROC curve. the length of the gallbladder in longitudinal scanning with a description of its wall regularity, and the thickness of the gallbladder in longitudinal scanning with a description of its wall regularity. Abnormal gallbladder findings were defined as follows: (1) gallbladder length evaluated as less than the normal lower limit for age (15 mm) [12]; (2) gallbladder thickness evaluated as greater than the normal upper limit for age (3 mm) [12]; or (3) absence of the gallbladder [13]. Ultrasonographic examination of patients was performed by an experienced ultrasound doctor.

### Statistical analysis

Descriptive results were expressed as number (percentage), mean ± standard deviation (mean ± SD), or median (interquartile range, IQR) (for data that were not normally distributed). For quantitative data, chi-square tests or Fisher’s exact tests were performed to detect the statistical significance of differences between groups, while analysis of variance (ANOVA), t tests, and Wilcoxon tests were used for continuous variables. Binary univariate and multivariate logistic regression analyses were performed for each variable and are presented herein. The diagnostic performance was expressed as sensitivity, specificity, positive predictive value (PPV), negative predictive value (NPV), and accuracy (percentage of correctly identified patients), and all were expressed as percentages. The cut-off values for optical clinical performance (best sensitivity and specificity simultaneously) of individual parameters and the overall accuracy of the scoring system were determined from the ROC.

A decision tree (DT) was constructed using the *R* package *rpart*, and a DT plot was drawn using the rattle package. In short, the root node or the first question was “Was γGT > 184 U/L in the patient”? In the subsequent classification trees, “no” indicated a branch to the right, while “yes” represented a branch to the left. The terminal nodes were used to predict BA or non-BA. DT was built for prediction using the RF package with 500 regression trees. The results were considered significant if the *p* value was ≤ 0.05. Statistical analysis was performed using SPSS Statistics 20.0 and SPSS Modeller 18 software for Windows (SPSS Inc., Chicago, IL, USA).

## Results

### Demographic, laboratory, and clinical characteristics of the study participants

A total of 148 infants between December 2008 and December 2018 met the eligibility criteria and were retrospectively enrolled. Of these, 52 infants (35.1%) were diagnosed with BA, and the remaining 96 infants (64.9%) had cholestasis due to causes other than BA (non-BA). The mean age was 48 ± 10 days in the BA group and 44 ± 11 days in the non-BA group (*P* = .02). The majority of non-BA patients were male (55.2%), although the sex distribution was nearly equal in the BA (males, 46.2%) group (*P* = .31). Stool colour, DBIL, DBIL/TBIL, γGT, gallbladder abnormalities, and positive TC signs were significantly different between the BA and non-BA groups (*P <* .01), whereas the two groups did not show any differences in ALT, AST, TBIL, LDH, ALP, or hepatomegaly (*P* > .05). Data for the other characteristics, including clinical findings (stool colour), laboratory findings (ALT, AST, TBIL, DBIL, DBIL/TBIL, γGT, LDH, and ALP), and ultrasonography (hepatomegaly, gallbladder abnormalities, and TC sign) are also listed in Table 1.

**Table 1:** Clinical, laboratory, and ultrasonographic characteristics of the model design cohort

Characteristics	BA (*n* = 52)	Non-BA (*n* = 96)	*P* value
Age (days)	48 ± 10	44 ± 11	0.02
Male sex (%)	24(46.2)	53(55.2)	0.31
Clay-like stool (%)	10(43.5)	7(13.2)	< 0.01
ALT(U/L)	173.7 ± 112.2	165.8 ± 155.6	0.78
AST(U/L)	284.2 ± 163.8	244.0 ± 212.8	0.28
TBIL (µmol/L), median (IQR)	165(130,200)	159(80.5,211)	0.06
DBIL (µmol/L), median (IQR)	116(95.5,154)	97(32.5,149.5)	0.01
DBIL/TBIL (%), median (IQR)	74.6(67.7,82.6)	66.9(54.4,78.2)	< 0.01
γGT (U/L), median (IQR)	447(206,822)	165(104,319)	< 0.01
LDH (U/L),median (IQR)	410(322.5,649)	485(336.5,809)	0.15
ALP (U/L)	616.7 ± 248.1	501.6 ± 275.9	0.07
TBA (µmol/L)	157.1 ± 63.0	124.1 ± 70.5	0.17
Hepatomegaly (%)	2(10.5)	5(8.6)	0.80
Abnormal gallbladder (%)	34(91.9)	2(2.8)	< 0.01
TC sign positive (%)	30(81.1)	12(20.7)	< 0.01

*BA* Biliary atresia, *Non-BA* Non-biliary atresia, *ALT* Alanine transaminase, *AST* Aspartate transaminase, *IQR* Interquartile range, *TBIL* Total bilirubin, *DBIL* Direct bilirubin, *γGT* Gamma glutamyl transpeptidase, *LDH* Lactate dehydrogenase, *ALP* Alkaline phosphatase, *TBA* Total bile acid, *TC* Triangular cord, *ANOVA* Analysis of variance

### Univariate logistic regression analysis of variables significantly associated with BA

Univariate and multivariate logistic regression analyses were performed to determine independent variables associated with BA. Statistically significant differences in variables, including stool colour, DBIL, DBIL/TBIL, γGT, gallbladder, and TC signs, were identified between the BA and non-BA groups (Table 1). The γGT and TC signs showed a medium independent prediction property with AUC > 0.7. However, the AUCs for stool colour, DBIL, DBIL/TBIL, and gallbladder findings were < 0.7 (Table 2).

**Table 2:** Diagnostic parameters in comparisons between the BA and non-BA groups in the model design cohort

Parameter	AUC	*p* value	Cut-off	Sensitivity	Specificity	PPV	NPV	Accuracy
Clay-like stool	0.651	< 0.01		43.5%	86.8%	58.8%	78.0%	73.7%
DBIL (µmol/L)	0.638	0.01	53.5	94.2%	34.4%	43.8%	91.7%	55.4%
DBIL/TBIL (%)	0.671	< 0.01	63.4	80.8%	45.8%	44.7%	81.5%	58.1%
γGT (U/L)	0.787	< 0.01	184.0	76.9%	75.0%	62.5%	85.7%	75.6%
Abnormal gallbladder	0.660	< 0.01		91.9%	48.4%	51.5%	90.9%	64.6%
TC sign (mm)	0.754	< 0.01	3.6	63.0%	90.0%	94.4%	94.4%	70.2%

*BA* Biliary atresia, *Non-BA* Nonbiliary atresia, *AUC* Area under the receiver operating characteristic curve, *PPV* Positive predictive value, *NPV* Negative predictive value, *DBIL* Direct bilirubin, *TBIL* Total bilirubin, *γGT* Gamma glutamyl transpeptidase, *TC sign* Triangular cord sign

### Establishment and validation of the logistic regression-based nomogram in predicting BA

A nomogram to predict BA was developed on the basis of multivariable logistic regression analysis using the six factors that were identified to be significantly different between the BA and non-BA groups, namely, stool colour, DBIL, DBIL/TBIL, γGT, abnormal gallbladder findings, and TC sign positivity. We found that stool colour, γGT levels, abnormal gallbladder findings, and TC sign positivity were significantly associated with BA (*P* = < .01), whereas DBIL and DBIL/TBIL were not (*P* > .05); thus, these four factors were used as predictors to build the nomogram prediction model for BA. The relationship between these factors and BA was assessed using multivariate logistic regression; the resulting data are presented in Table 3.

**Table 3:** Predictors of BA in patients with neonatal cholestasis selected in the model design cohort^a^

Variable	β	Odds ratio^b^	*p* Value
Clay-like stool	1.26	3.53 (1.61,15.89)	< 0.01
γGT	2.80	16. 49(0.99,0.99)	0.01
DBIL	>-0.01	0.99 (0.99,1.00)	0.31
DBIL/TBIL	-0.14	0.99 (0.95,1.02)	0.41
Abnormal gallbladder	1.72	5.57 (1.28,24.31)	0.02
TC sign positive	2.45	11.53 (3.26,40.69)	< 0.01

*BA* Biliary atresia, *γGT* Gamma glutamyl transpeptidase, *DBIL* Direct bilirubin, *DBIL/TBIL* Direct bilirubin/total bilirubin, *TC Sign positive* Triangular cord sign positive

^a^Assessed by using multivariable logistic regression model

^b^Numbers in parentheses are 95% confidence intervals logistic regression

### Establishment of the DT model in predicting BA

**Fig. 1:**
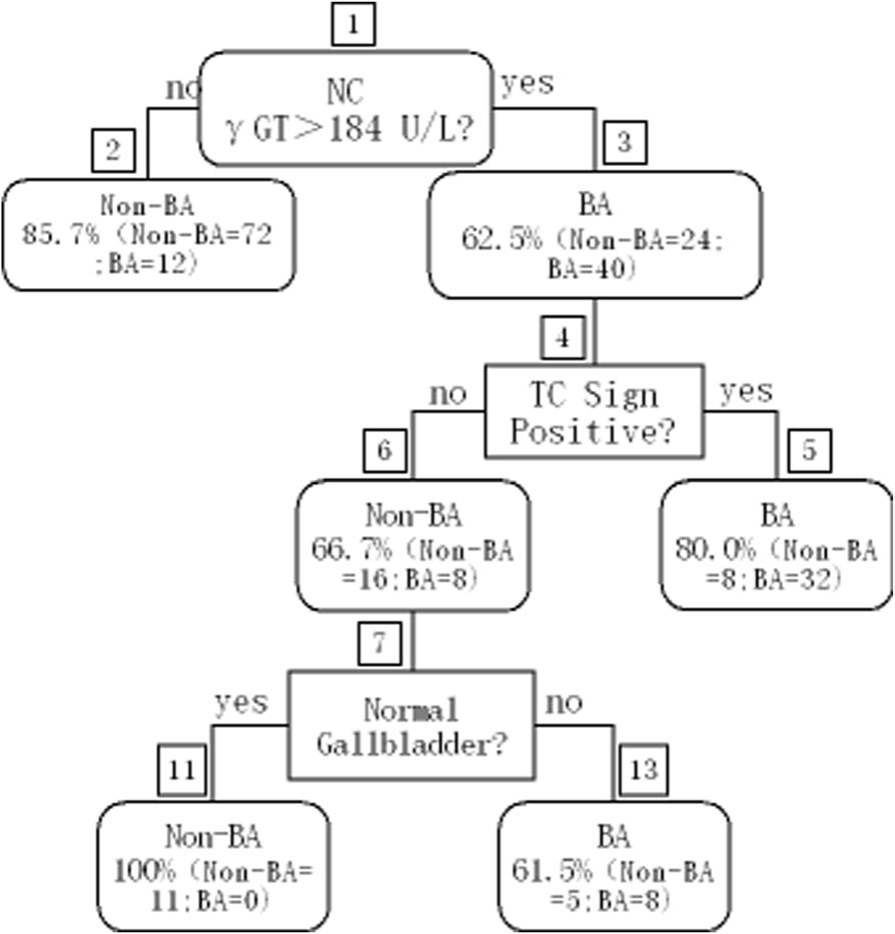
Decision tree (DT) for the prediction of biliary atresia (BA) using gamma glutamyl transpeptidase (γGT) concentration, triangular cord (TC) sign, and gallbladder findings in patients with neonatal cholestasis (NC). The DT included five variables and was implemented in the general R package rpart. The root node query was as follows: Was γGT > 184 in the patient? In the classification tree, “no” resulted in a branch to the right. A total of nine terminal nodes were generated for the DT model. Non-BA, non-biliary atresia

 The DT for the prediction of BA included four study variables: stool colour, γGT, abnormal gallbladder, and TC sign positivity. For the establishment of the DT model, the first question, also known as the root node, was (1) Was γGT > 184 U/L in the patient? In the classification tree, “no” represents a branch to the left. Infant patients who met this criterion were classified as non-BA. If the answer was “yes,” the second question was (2) Was the TC sign positive? Infant patients who met this criterion were classified as BA. For patients who did not meet the criteria, the tree further queried (3) Was the gallbladder normal? If the answer was “no,” the patients were classified as BA. If the answer was “yes,” the patients were classified as non-BA (Fig. 1). The DT model could discriminate BA with 77.0% sensitivity and 86.0% specificity and an overall diagnostic accuracy of 83.0%.

### Validation of the BA diagnostic model

To verify the applicability of this model, a second cohort of infants with cholestatic liver disease was tested, which included infants with BA (*n* = 10) and those with other infantile cholestatic liver diseases (*n* = 11). The mean age was 52 ± 8 days in the BA group and 45 ± 11 days in the non-BA group (*P* = .21). The majority of BA patients were male (60%), and a few non-BA patients were male (36.4%) (*P* = .28). Data for other characteristics, including γGT, abnormal gallbladder and TC sign positivity, are also listed in Table 4.

**Table 4:** Clinical, laboratory, and ultrasonographic characteristics of the model validation cohort

Characteristics	BA (*n* = 10)	Non-BA (*n* = 11)	*p* value
Age (days)	52 ± 8	45 ± 11	0.21
Male sex (%)	6(60.0)	4(36.4)	0.28
γGT (U/L), median (IQR)	338(223.0,1094.5)	97(78,165)	< 0.01
Abnormal gallbladder (%)	8(80.0)	6(54.5)	0.23
TC sign positive (%)	9(90.0)	1(9.1)	< 0.01

*BA* Biliary atresia, *γGT* Gamma glutamyl transpeptidase, *IQR* Interquartile range, *TC Sign positive* Triangular cord sign positive

Only one of the patients with BA (1/10) was wrongly categorized into the non-BA group, the γGT concentration of the patient was 134 U/L classified as non-BA according to Fig. 1 node 2, and we had no opportunity to use other indicators because there was no branch below node 2. The TC sign was positive, and the gallbladder was abnormal. One of the patients without BA (1/11) was wrongly categorized into the BA group. The γGT concentration of the patient was 192 U/L, classified as BA. It is possible that the γGT concentration of the patient was nearly the cut-off. The model could discriminate BA with 90.0% sensitivity and 90.9% specificity and an overall diagnostic accuracy of 90.5%. The AUC was 0.91, which was better than those of γGT concentration, gallbladder abnormality, and TC sign positivity alone (0.86 0.63, and 0.86).

## Discussion

Accurate diagnosis of BA using existing diagnostic approaches is challenging primarily because of the overlapping features between BA and other forms of NC attributable to different causes. Moreover, the current diagnostic methods may involve radiation exposure or are costly, highly technical, and invasive. We developed a predictive and simple diagnostic model to differentiate BA from other causes of NC at an early stage. Our diagnostic model was composed of three variables based on simple laboratory and imaging findings, which can be obtained cost-effectively, quickly, and noninvasively without exposure to radiation. Validation of the model revealed its high discrimination ability, which was better than those of γGT concentration, gallbladder abnormalities, and TC signs positive alone. Therefore, this model can help clinicians easily and promptly diagnose BA before 60 days.

Early accurate diagnosis of BA is critical for timely intervention with KP to restore bile flow and slow down the progression of this disease in infants [4, 14]. The performance of KP before 60 days of age is known to have better outcomes, and the survival rates with native liver decreased when the age at surgery increased [15]. Therefore, we excluded patients older than 60 days from the study. To our knowledge, this is the first study conducted with infants within 60 days of age.

The current preoperative variables used for the diagnosis of BA primarily include laboratory indices, such as bilirubin [16], DBIL [17], and γGT [6] concentrations. Some studies have reported using γGT to diagnose BA [6, 18], although the cut-off values varied across these studies. Rendon-Macias revealed that a γGT level > 250 U/L had high diagnostic value for BA [18], and Tang reported that a γGT level > 300 U/L had high diagnostic value [6]. Since the reference value of neonatal γGT level is greatly affected by age, an increasing number of recent studies have suggested that different cut-off values should be used to evaluate infants at different ages [19]. In this study, the cut-off value of γGT was 184 U/L, and the difference from other studies could be attributed to the fact that we selected infants within 60 days of age, which represents a younger population than that reported in previous studies [6, 18].

There are some other parameters that require consideration in this regard. Some indicators we think important, such as clay-like stools, were not included in the model. We found that the odds ratio of clay-like stool was less than that of other stools, perhaps because the early stools of children with BA may show yellow, yellow‒green, or other normal infant stool colours. However, when the bile excretion channel is partially obstructed, stool can appear pale yellow, and the degree of obstruction increases with age, finally resulting in clay or grey‒white stools. The patients we included had early-stage BA. This may be the reason that clay-like stool is not included in the model. Ultrasonography can help rule out biliary malformations such as congenital choledochocoele. It offers the advantages of simplicity, non-invasiveness, cost-effectiveness, and dynamic observation. This is a routine inspection item for children with NC [20, 21]. The TC sign is a very important diagnostic feature [5]. Many studies have reported that the TC sign shows high specificity for diagnosing BA [5, 22]. However, in younger infants, the TC sign may not have completely formed, making its evaluation unclear; thus, the positive rate of the TC sign varies greatly in different age groups [5]. A TC sign > 3.4 mm [23] or > 4 mm [24] has been reported to show high diagnostic specificity. In this study, the cut-off TC sign was 3.6 mm. Children with BA often show poor development of the gallbladder or cystic duct, morphological changes, and retraction of the gallbladder. Our study found that the sensitivity and specificity of TC sign positivity were 63.0% and 90%, respectively, and those of gallbladder abnormalities were 91.9% and 48.4%, respectively, in accordance with the results reported by Yoon [24]. The model was accurate for the diagnosis of BA and has potential for clinical application.

In addition to abdominal ultrasound, several medical imaging techniques have been used for screening BA, including cross-sectional magnetic resonance imaging (MRI), hepatobiliary scintigraphy, cholangiopancreatography (MRCP) [25], duodenal tube test (DTT) and liver biopsy [26]. Yang reported that serum matrix metalloproteinase-7 (MMP-7) may be a reliable biomarker for BA, with high sensitivity (98.7%) and specificity (95.0%) [27]. However, while MMP-7 evaluations may be possible in paediatric liver disease centres, for most clinicians, the MMP-7 level is not a primary evaluation parameter, since many clinicians may be unaware of its significance and most hospitals may not be equipped to perform MMP-7 assays.

Most recently, Kim et al. established an ultrasonography and hepatobiliary scintigraphy-based score for the diagnosis of BA in infants with jaundice and reported good discrimination ability (AUC = 0.98) [28]. However, this score required hepatobiliary scintigraphy, involved exposure to radiation and was technically difficult. El-Guindi developed a diagnostic score for BA that included clinical, laboratory, ultrasonographic, and histopathological parameters, which showed a high accuracy rate of 98.8% in predicting BA [8]. However, liver biopsy is invasive and technically difficult. Thus, existing diagnostic methods appear to have a number of limitations: they are costly and/or involve exposure to radiation, technical difficulty, or highly invasive procedures.

Although our study offered useful information about the value of the nomogram for diagnosing BA, it has a number of limitations that must be acknowledged. First, the model was established retrospectively, and a selection bias may exist as a result. Second, we aimed to discriminate BA from NC in the early stage when KP was feasible, so only infants within 60 days of age were included. Thus, the model may not be appropriate for infants aged > 60 days. Third, the sensitivity of model development was 77.0%, which means that approximately 23.0% of patients (BA with γGT less than 184 U/L) will be mistaken for non-BA because the TC sign and gallbladder cannot be used in these patients. Therefore, the sensitivity of the model should be further improved in the future. For example, different γGT thresholds should be analysed to improve sensitivity, or a diagnosis score system should be developed that combines all indicators. Furthermore, we initially reviewed the data for more than 1000 patients, but most of them did not have BA, while others had BA but were more than 60 days of age; thus, these patients were excluded from our study. Finally, this model was only based on regular liver function and abdominal ultrasonography markers, and other biomarkers were not assessed. Thus, future studies should recruit more infants to validate the model.

## Conclusion

By using liver function and abdominal ultrasonography data, we developed a simple and easily applicable model that showed moderate discrimination ability for the diagnosis of BA. This model is only for patients younger than 60 days. For patients aged > 60 days, the model may not be appropriate and may need to be combined with other indicators. However, the model may facilitate prediction of the risk of BA for patients younger than 60 days.

## Data Availability

The data that support the findings of this study are available from the corresponding author on reasonable request.

## Abbreviations

BA: Biliary atresia
NC: Neonatal cholestasis
DT: Decision tree
γGT: Gamma glutamyl transpeptidase
TC sign: Triangular cord sign
ROC: Receiver operating characteristic
AUC: Area under the receiver operating characteristic curve
KP: Kasai portoenterostomy
DBLL: Direct or conjugated bilirubin
TBIL: Total serum bilirubin
IOC: Laparotomy and intraoperative cholangiography
DBIL/TBIL: Direct bilirubin/total bilirubin ratio
ALT: Alanine transaminase
AST: Aspartate transaminase
ALP: Alkaline phosphatase
TBA: Total bile acid
LDH: Lactate dehydrogenase
IQR: Interquartile range
ANOVA: Analysis of variance
PPV: Positive predictive value
NPV: Negative predictive value
MRI: Magnetic resonance imaging
MRCP: Cholangiopancreatography
DTT: Duodenal tube test
MMP-7: Matrix metalloproteinase-7
